# Circulating Tumor DNA Using Tagged Targeted Deep Sequencing to Assess Minimal Residual Disease in Breast Cancer Patients Undergoing Neoadjuvant Chemotherapy

**DOI:** 10.1155/2020/8132507

**Published:** 2020-01-22

**Authors:** Gabriella Cirmena, Anna Garuti, Marilena De Mariano, Simona Coco, Lorenzo Ferrando, Edoardo Isnaldi, Valentina Barbero, Piero Fregatti, Lucia Del Mastro, Fabio Ferrando, Roberta Gonella, Alessandro Garlaschi, Daniele Friedman, Alberto Ballestrero, Gabriele Zoppoli

**Affiliations:** ^1^Department of Internal Medicine, University of Genoa, 16132 Genoa, Italy; ^2^Lung Cancer Unit, Ospedale Policlinico San Martino, 16132 Genoa, Italy; ^3^Ospedale Policlinico San Martino, 16132 Genoa, Italy; ^4^Department of Surgical Sciences and Integrated Diagnostic DISC, 16132 Genoa, Italy

## Abstract

In breast cancer patients undergoing neoadjuvant chemotherapy before surgery, there is an unmet need for noninvasive predictive biomarkers of response. The analysis of circulating tumor DNA (ctDNA) in particular has been the object of several reports, but few of them have studied the applicability of tagged targeted deep sequencing (tTDS) to clinical practice and its performance compared with droplet digital PCR (ddPCR). Here, we present the first results from an ongoing study involving a prospectively accrued, monocentric cohort of patients affected by invasive breast cancer, undergoing neoadjuvant chemotherapy followed by surgery with curative intent as per clinical practice. A pretreatment tumor biopsy and plasma samples were collected before and during treatment, after surgery, and every six months henceforth or until relapse, whichever came first. Pretreatment biopsies were sequenced with a 409-gene massive parallel sequencing (MPS) panel, allowing the identification of target mutations and their research in plasma by tTDS and ddPCR as a complementary approach. Using tTDS, we demonstrated the presence of at least one deleterious mutation in all the relapsed cases we studied (*n* = 4), with an average lead time of six months before clinical relapse. The association with ddPCR was suboptimal, and only one relapsed patient could be identified with such method. tTDS shows potential as an early noninvasive method for the detection of MRD in BC patients.

## 1. Introduction

Breast cancer (BC) is the most common type of cancer in women worldwide, and approximately 30% of patients initially diagnosed with early-stage cancer will eventually develop metastatic disease [1]. Therefore, there is an urgent need for biomarkers to identify minimal residual disease (MRD) and to better assess the risk of relapse during patients' follow-up. Circulating tumors cells (CTCs) were approved by the Federal Drug Agency (FDA) for the prognostic stratification of early breast cancer patients, but the sensitivity of this method is relatively low and the retrieval of CTC is not a trivial task [2, 3]. Recently, the detection of circulating tumor DNA (ctDNA) in the blood has showed promise in providing prognostic and predictive information for the clinical management of breast cancer patients [4–6]. The use of ctDNA has advantages over the use of tissue biopsies due to its availability with minimally invasive procedures, and the opportunity to obtain multiple samples over several time points. Therefore, sensitive assessment of the presence of ctDNA may represent an ideal biomarker for the purposes outlined above [7]. Efforts to detect ctDNA have intensified in recent years [8–10], and the advent of massively parallel sequencing (MPS) has provided unprecedented opportunities as well as threats in such regard [11–13].

On the one hand, ctDNA tracking over time can constitute the basis of advanced personalized treatment and could be used to monitor the presence of MRD. On the other hand, benchmarking and optimization of new technical platforms for ctDNA detection are mandatory before its introduction into clinical practice. First of all, the control of various parameters, from blood collection to isolation of circulating DNA, has a significant impact on the quality and accuracy of the data. Tumor-specific digital droplet polymerase chain reaction (ddPCR) assays are highly accurate, but require the design and optimization of personalized assays, an expensive, time-consuming, and not always successful step. Furthermore, genetic aberrations of metastatic disease may differ from those found in the primary tumor [14]. Tagged targeted deep sequencing of plasma DNA (tTDS), a novel proprietary method now under active development [15, 16], could provide a better alternative for high-throughput analysis of ctDNA compared with ddPCR and may overcome limitations of initial tumor tissue assessment allowing for the direct identification of several low-frequency ctDNA mutations.

Here, we present the results of tTDS using Oncomine™ Breast v2 cfDNA Assays (Thermo Fisher Scientific™) in BC patients undergoing neoadjuvant chemotherapy (NACT). The aims of the present study were (1) to demonstrate the feasibility of obtaining circulating cell-free DNA (ccfDNA) from plasma samples suitable for NGS by performing appropriate quality controls and (2) to develop an optimized workflow for mutation tracking in serial plasma samples to predict treatment response and/or early relapse in BC patients undergoing NACT followed by surgery with curative intent as per clinical practice.

## 2. Patients and Methods

### 2.1. Patients

Inclusion criteria were: histological diagnosis of invasive breast cancer with indication to NACT as per clinical practice, completion of at least 85% of NACT; availability of enough material from the diagnostic biopsy at diagnosis for NGS assessment; availability of plasma samples at the specified time points, i.e., basal, half treatment completed, before surgery, at 12 weeks, 24 weeks, 1 year after surgery, and every 24 weeks until 24 months of follow-up or upon relapse; willingness to participate; and written informed consent. Exclusion criteria were: death from non-cancer-related causes within the first 12 months after surgery; unwillingness or inability to participate; multicentric/bilateral disease; pT1mic/pN0 at surgery, due to the scarcity of data concerning the prognostic value of such therapeutic result; diagnosis of advanced disease within six months of diagnosis; and refusal to participate or consent withdrawal.

BC patients undergoing NACT were recruited between 2014 and 2018. All patients underwent pretreatment clinical and radiological staging before treatment, as per clinical practice. Baseline radiological staging of all patients was obtained by magnetic resonance and whole-body CT, and expressions of Ki-67, estrogen receptor (ER), progesterone receptor (PR), and HER2 status were assessed locally as per SIAPEC/ASCO/CAP criteria. Surgical and histopathological findings such as tumor subtype as well as clinical data of direct relevance for our study and prespecified per protocol were also recorded. After completion of NACT, patients underwent surgery. The histopathological findings at surgery were compared with pretreatment staging. Pathological complete response (pCR) was defined as the complete absence of invasive tumor in the primary site and excised lymph nodes. *In situ* neoplasia (Tis) was not considered as invasive disease.

### 2.2. Extraction of DNA from Biopsies and PBMCs

DNA from formalin-fixed paraffin-embedded (FFPE) breast tissue samples was extracted using the Maxwell® RSC DNA FFPE Kit with the Maxwell® RSC Instrument (Promega Corporation, Madison, USA) according to the manufacturer's instructions. Germline DNA from peripheral blood mononuclear cells (PBMCs) was extracted using the Maxwell® 16 Blood DNA Purification Kit according to the manufacturer's instructions.

The concentration and purity of DNA samples were measured using both a NanoDrop™ 2000 spectrophotometer (Thermo Scientific™) and a Qubit™ dsDNA HS Assay Kit (Invitrogen™) designed for use with the Qubit™ 2.0 Fluorometer (Invitrogen™).

### 2.3. Mutational Analysis by MPS on Biopsy FFPE and PBMCs

The Comprehensive Cancer Panel™ (CCP, IonAmpliSeq™ Comprehensive Cancer Panel™, Thermo Fisher Scientific™) was used to identify target mutations in exonic regions of 409 cancer-related genes. Four libraries were created using 40 ng from both FFPE DNA from diagnostic biopsies and germline DNA samples as per manufacturer's specification [17] Libraries were quality-checked on an Agilent™ TapeStation. The Thermo Fisher Scientific™ Ion Chef™ system was used for template preparation followed by sequencing on an Ion PGM™ System using Ion 318™ chips, one library per chip for FFPE DNA samples and four germline libraries per chip [18].

### 2.4. Isolation, Extraction, and Quantification of Circulating Cell-Free DNA (ccfDNA)

Blood samples were collected in BD Vacutainer® EDTA tubes (Becton Dickinson). To separate plasma, whole blood was processed within 2 hours by centrifugation for 10 min at 1600 ×g and collected in a new conical tube. Plasma was then recentrifuged for further 10 min at 1600 ×g. The resulting plasma was stored at −80° until DNA extraction.

As per manufacturer's specifications, 10 nanograms of ccfDNA were used for ddPCR experiments and 20 ng for tTDS experiments. To ensure that such quantities were obtained, we collected three 7.5 ml peripheral blood test tubes for each patient. We then proceeded to extract ccfDNA from 2 to 5 ml of plasma from each of the two tubes according to individual yield. The third was stored as back-up in case of extraction failure of the need for further experiments. For extraction, we used the QIAamp Mini Elute cfDNA Mini or Midi Kit (Qiagen, Hilden, Germany) on the QIAcube system, according to the manufacturer's instructions. DNA was eluted into 40–50 *μ*l Ultraclean water in DNA Lo Bind tubes and stored at −20°C. Isolated cfDNA was then quantified by Qubit™ dsDNA HS Assay Kit (Invitrogen™) designed for use with the Qubit™ 2.0 Fluorometer (Invitrogen™) and for evaluation of fragment size on High Sensitivity D1000 ScreenTape for use with the TapeStation 2200 (Agilent Technologies, Germany).

### 2.5. Mutational Analysis by ddPCR on ctDNA

The gene mutations identified by NGS were validated by droplet digital PCR (ddPCR) on ctDNA samples samples using a QX200 Droplet Digital PCR system (Bio-Rad Laboratories, Inc., Hercules, CA, USA). Reactions were performed in 24 *μ*l volumes using 12 *μ*l ddPCR 2x Supermix (Bio-Rad Laboratories, Inc.), 1 *μ*l of 20x assay mix (9*μ*m primers, 5*μ*m TaqMan mutant Probe (Bioclarma S.r.l. Research and Molecular Diagnostics, Torino, Italy), and 2.5–10 *μ*l of ctDNA with a final concentration of 10 ng.

For each sample , 20 *μ*l of PCR reaction and 70 *μ*l of droplet generation oil were dispensed into specific wells of a DG8 cartridge and then loaded in the QX100 droplet (Bio-Rad Laboratories, Inc.). Then, 50 *μ*l of droplets were then transferred into the wells of a 96-well PCR plate, sealed, and loaded into the Mastercycler nexus gradient thermal cycler (Eppendorf, Hanburg, Germany) [19]. After PCR was completed, the plate was loaded into the droplet reader (Bio-Rad Laboratories, Inc.) to acquire the droplets. The data were analysed using the proprietary QuantaSoft software (Bio-Rad Laboratories, Inc.). Each PCR plate included a human reference DNA (Promega corporation, Madison, WI) as wild-type control (WTC), a synthetic DNA specific for each mutation ( Bioclarma S.r.L.) as positive control (PC), and a nontemplate control (NTC). Each ctDNA sample was run in duplicate, and the allele fraction was calculated as merged of replicates. For each mutation, the threshold was set manually based on signals of NTC, WTC, and PC. A mutation was only considered to be present if three or more FAM positive droplets were detected [20, 21].

### 2.6. Mutational Analysis by tTDS on FFPE Biopsy and ctDNA

We used the next-generation sequencing Oncomine™ Breast v2 cfDNA Assays (Thermo Fisher Scientific™) which is designed, under manufacturer's specifications, to detect somatic mutations in plasma down to a limit of detection (LOD) of 0.1% [22] in genes relevant to solid tumors. In the commercial panel, the following genes are investigated: hotspot genes (i.e., with mutations detectable only in already known regions) are AKT1, EGFR, ERBB2, ERBB3, ESR1, FBXW7, KRAS, PIK3CA, SF3B1, and TP53 (∼152 hotspots). Copy number genes (CNVs) included in the kit are CCND1, ERBB2, and FGFR1. TP53 has an 88% in silico coverage of the exonic regions. The library size was checked using the Agilent High Sensitivity DNA Kit by TapeStation 2200 instrument, and library concentration was evaluated with a Qubit® 2.0 Fluorometer using Qubit™ dsDNA HS Assay Kit. Each barcoded library was diluted to 50 pM concentration for template preparation on an Ion Chef System and sequenced on an Ion 540™ chip on the Ion S5 System to obtain a coverage ≥25,000x, as per manufacturer's specifications.

### 2.7. Power Considerations, Data Processing, and Statistical Analyses

Assuming an actionable mutation could be found in plasma before surgery in 70% of NACT patients without pCR and <10% of patients with pCR, with a two-sided *α* = 0.05 and 1 − *β* = 0.90, further assuming a pCR rate of 30%, we could reject the null hypothesis of finding ctDNA in 50% of patients regardless of pCR by analysing 10 patients undergoing NACT, with a 70/30 ratio (3 with pCR and 7 without). Accounting for the failure of obtaining and/or properly analysing the required samples at all time points of 50%, and a refusal to participate or consent withdrawal of 30%, at least 30 patients needed to be screened to proceed to the required analyses.

For identification and interpretation of genetic variants, we matched somatic DNA from biopsies and germline DNA sequencing results, in order to identify truncal, high allelic frequency somatic, and pathogenic mutations. For target mutation selection, in case of multiple variants fulfilling these requirements, we chose the one with the highest variant allelic frequency (VAF) and the greatest coverage. Moreover, we removed variants in genes with reported mutational sequencing in TCGA (provisional version, last accessed on cBioPortal in 2017) <3%.

We used the Ion Reporter® software for mutational analysis by MPS on biopsy FFPE and PBMCs, workflow version 5.10 AmpliSeq CCP v1.1—Tumor-Normal pair, with functional filters as follows: confident somatic variants included; location in exonic; 0.05 ≤ allele ratio ≤ 1.0; variant type in SNV, INDEL, MNV, LONGDEL, CNV; UCSC common SNPs = not included.

Oncomine™ panels on liquid biopsy samples and tumor biopsies were analysed first with the Torrent Suite Software version 5.4, using the ctDNA Variant Caller plug-in with parameters optimized for “tagseq_cfDNA and tagseq_ffpe, TS version 5.10” application and later with the Ion Reporter® software, with the following workflows: version 5.10 Oncomine TagSeq Breast v2 Liquid Biopsy workflow 2.1—Single Sample, functional filter Oncomine 5.10, and version 5.10 Workflow Oncomine TagSeq Breast v2 Tumor workflow 2.1—Single Sample following standard user guides.

## 3. Results

### 3.1. Demographics

We collected pretreatment formalin-fixed, paraffin-embedded tumor biopsies as well as plasma samples from 38 early IBC patients consecutively undergoing NACT followed by surgery with curative intent. Of these patients, 29 donated plasma beyond T0 and 25 had an available pretreatment biopsy, 15 patients had all their samples collected or relapsed at least six months after surgery. Two patients withdrew from the study. Three patients were metastatic at diagnosis. Samples from the first 10 patients who completed at least 24 months of follow-up or at relapse were analysed. The demographics and pathological data characteristics of the study cohort are presented in Table 1.

**Figure 1:**
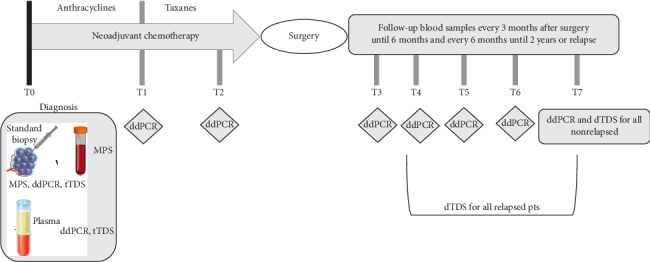
Diagram workflow of the planned sample collection and analyses: at diagnosis, tumor biopsies of patients presenting with invasive breast cancer and indication for NACT were analysed by MPS to identify somatic tumor-specific mutations and then confirmed by ddPCR and tTDS. Mutations in plasma were analysed by ddPCR at baseline, half treatment completed, before surgery, at 12 weeks, 24 weeks, 1 year after surgery, and every 24 weeks until 3 years of follow-up or upon relapse. tTDS was assessed at baseline on biopsies and plasma, as well as at relapse or last follow-up time point.

MPS, ddPCR, and tTDS were performed on DNA extracted from 10 tumor biopsies. MPS was also performed on germline DNA. Circulating tumor DNA was successfully extracted in all collected plasma samples. ctDNA was analysed by digital droplet PCR and tTDS. A diagram describing the study is reported in Figure 1.

### 3.2. Individualized Driver Mutations by MPS Analysed by ddPCR for Mutation Tracking

Ten FFPE tissue biopsies and matched germline DNA were successfully extracted. The median yield was 140.0 ng/*μ*l (min. 72.3; max 267.7). All samples were successfully amplified with the Comprehensive Cancer Panel™. The sequencing metrics for FFPE sequencing runs were as follows: median mapped reads 5,506,603 (IQR 4,432,976–5,6080,223), average base coverage median 316x (IQR 245–367x), and uniformity base coverage (meant as the proportion of bases read deeper than 20% of overall coverage) median percent 91.0 (IQR 85.8–92.8). We identified six somatic mutation variants according to our established selection criteria, in three different genes: TP53, PIK3CA, and GATA3, with VAF ranging from 6.4% to 29.7%. Such mutations were transversally validated in the same samples using ddPCR (see Table 2).

**Figure 2:**
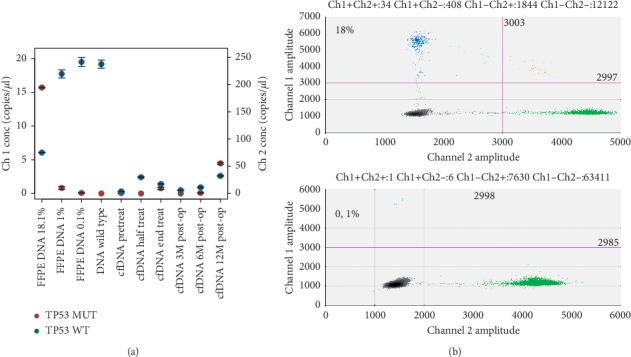
ddPCR anticipates clinical relapse in one patient with a TP53 mutation. (a) Assessment of target mutation by ddPCR during the monitoring on ctDNA in a patient (UPN1) relapsed at 12 months. TP53 p. Y205C c.614A>G was present at 6 months (T4) after surgery, anticipating the patient's clinical relapse by six months. (b) ddPCR scatterplot of target mutation (*y*-axis) vs. wild type (*x*-axis) on FFPE biopsy at diagnosis (upper plot) and on ctDNA at T4 (lower plot).

The ddPCR assays we validated were then used to track the presence of those mutations in serial plasma samples, since some of the selected mutations were not covered by Oncomine Breast v2 cfDNA Assay [TP53 c.614A>G; TP53 c158G>A; GATA3 c.1223_12124insA]. MPS and ddPCR analyses had a good level of agreement in baseline tumor DNA concerning the assessment of mutational VAF, except for one patient (UPN4). Indeed, mutations in ctDNA matching those selected by MPS were detected in 80% (4 of 5; 95% CI, 37.5% to 96.3%) of baseline plasma samples (see Table 3). In none of the five cases, we could detect the selected mutations after the first three months of chemotherapy or before surgery. In one relapsed case, we observed the disappearance of mutated TP53 in ctDNA after surgery and its detectability (0.11 copies/*μ*l) at 24 weeks (T4), anticipating distant relapse by six months (see Figure 2). Of interest, in two patients who have not relapsed by the time of the present work, we detected the selected mutations by ddPCR in plasma at 24 months, without signs of invasive disease as per clinical practice.

### 3.3. Driver Mutations Analysed by MPS and tTDS on FFPE Tumor Samples at Diagnosis

Ten FFPE tissue biopsies previously analysed by MPS were resequenced by tTDS, using the commercial Oncomine TagSeq Breast v2 Liquid Biopsy panel. We were able to generate libraries from 15 ng to 20 ng of FFPE DNA, with an average of 17.6 ng per sample, and successfully amplified for sequencing of all the assessed samples. FFPE samples were sequenced at a median read coverage of 27,764x (IQR 21,390x–38,557x), with uniquely tagged molecules covered at a median of 1,719x (IQR 1,061.5–2,300). The median limit of detection, as assessed by the manufacturer's software, was 0.60 % (IQR 0.64–0.67%). Concerning regions covered by the designs of both MPS and tTDS, these methods showed a very good level of agreement in baseline tumor DNA assessment, with tTDS identifying all mutations selected by MPS (see Table 4). Of note, using tTDS, we correctly identified the copy number gain of ERBB2 in one patient, as expected by IHC results. Additionally, several low VAF mutations were observed by tTDA, due to its higher sensitivity compared with MPS. These however will not be covered in the present work since we did not perform transversal validation of their actual presence.

### 3.4. Tracking Mutations in ctDNA with tTDS to Identify Minimal Residual Disease (MRD) and Anticipate Relapse

**Figure 3:**
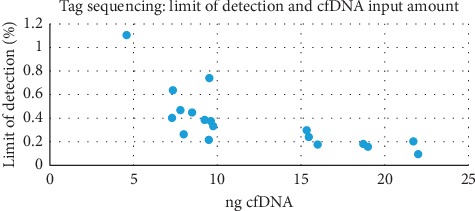
Scatterplot highlighting the correlation between cfDNA input amount (*x*-axis) and limit of detection (LOD, *y*-axis).

An average of 12.2 ng (range 4.6–21.7 ng) could be extracted and used for tTDS library preparation starting from 2 to 5 mL plasma samples. To assess the validity of our tTDS analyses, to each run we added 20 ng of Horizon Control Multiplex I cfDNA Reference Standard, which is designed to include known somatic mutations covered by our tTDS panel at fixed concentrations of 5%, 1%, and 0.1%. To quantify libraries generated by Oncomine Breast v2 cfDNA, we followed the same recommendations as for FFPE samples (see Methods section). The sequencing metrics for cfDNA sample runs were as follows: median read coverage 30,240x (IQR 22,274.25–41,120.25x) and median coverage of individual tagged molecules (defined by the manufacturer as median molecular coverage) 2,094.5x (IQR 1,261.5–3,144.25x). The median LOD was 0.31% (IQR 0.19–0.45%) (see Figure 3).

Of the samples from the six patients who did not relapse, we could not detect any mutation by tTDS in four cases at the last available time point. In two of those cases, we detected ctDNA mutations in plasma samples, and one of the two cases had the same mutation (TP53 R248W) in both plasma and in the pretreatment FFPE specimen at very low VAF. Three relapsed patients out of four had detectable plasma mutations at diagnosis by tTDS, and in two of these three cases, the same mutations could be confirmed in tissue pretreatment samples. Patient UPN1, for whom a mutation in TP53 could be identified and tracked by MPS and ddPCR both at diagnosis and before relapse, could not be studied by tTDS due to the lack of coverage of that genomic region in the Oncomine Breast tTDS panel (see Table 5).

**Figure 4:**
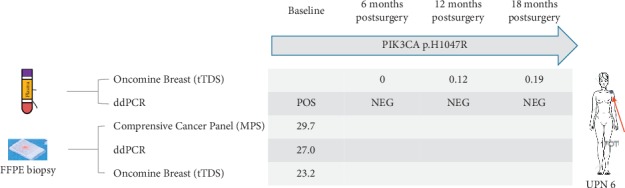
Comparison case in point of MPS, MPS, and tTDS for mutation tracking: PIK3CA mutation H1047R could be detected in plasma and in the FFPE pretreatment biopsy by MPS, ddPCR, and tTDS. However, ddPCR allowed the detection of such mutation only at baseline, whereas by tTDS we could track the presence of H1047R six months before relapse.

In one patient (UPN6), both the pretreatment FFPE biopsy and ctDNA harboured the same PIK3CA H1047R somatic mutation and also confirmed at baseline by ddPCR, but only tTDS could detect the reappearance of that mutation in plasma six months before (see Figure 4).

Finally, in one case (UPN8), we could detect mutations at several time points before relapse, but those were not identified in the pretreatment biopsy and varied through time. Interestingly, mutations in KRAS could be found in that case in multiple time points, albeit not in the same genomic position.

## 4. Conclusion

In the present work, we describe the results of a noninterventional, retrospective-prospective case-control study aimed at assessing the presence of mutations in plasma for MRD tracking in patients affected by BC, undergoing NACT for their disease as per clinical practice. To this purpose, we adopted two strategies: (i) targeted MPS of pretreatment FFPE biopsies with a 409-gene panel, followed by stringent selection of an individualized mutation to be assessed as a personalized marker in plasma over time using ddPCR; (ii) evaluation of the presence of mutations in ctDNA using a novel ultrasensitive targeted sequencing approach, namely, tTDS. Our effort allowed us to identify few mutations answering our criteria in half of our patients' group for ddPCR tracking. With this method, four out of the five patients for whom we designed a ddPCR assay presented with the selected mutation in plasma at baseline. In no case, the personalized tracker mutations were detected after three months of NACT or before surgery, independently from pCR. In only one relapsed case out of four, we could identify with certainty the reappearance of the marker mutation before clinical relapse, with a lead time of six months.

On the other hand, using a commercial tTDS panel—not designed specifically for MRD monitoring—we could detect at least one mutation in three out of four patients who relapsed, usually six months before clinical progression.

We did not have sufficient material left for analysis before surgery, and thus, a comparison with ddPCR cannot be done in this regard. Of interest, by the end of the study, two out of six nonrelapsed patients exhibited the persistence of ctDNA mutations, and these were observed both in plasma and in the pretreatment biopsy using tTDS.

Over the past few years, ctDNA tracking for MRD in BC has been performed with different strategies. Garcia-Murillas et al. [20] described an approach similar, in concept, to what we did. Specifically, however, they assessed snap-frozen pretreatment biopsies using a breast-specific, custom-designed MPS panel. Then, the authors went on designing one or more patient-specific ddPCR assays and used them for personalized monitoring of relapse. Their results were by far more successful than the ones presented in our work, possibly reflecting the context (a prospective clinical trial and the analysis of snap-frozen material), the used panel (a breast-specific one), the choice of multiple ddPCR-personalized probes for monitoring, and a larger cohort of patients. However, that approach was not followed by confirmatory publications, suggesting that, as we experienced, the design of patient-specific ddPCR assays is not an easy task in clinical practice.

Another case-control study [23] assessed the presence of genomic rearrangements in BC surgical specimens by low-pass whole-genome sequencing, and prospectively collected plasma samples were then tested in a time series for the presence of patient-specific translocations assessed by RT-PCR. In the reported work, the authors showed a high degree of sensitivity for the reappearance of genomic aberrations in relapsed patients. Such analysis however came at a high cost in terms of MRD marker personalization and seems of difficult transferability to clinical practice. Of interest however, in that report, the authors identified marker mutations in ctDNA of relapsed patients with a similar lead time to our cases, hinting that the release of appreciable quantities of ctDNA may anticipate clinical relapse by six months in many cases. In that work as in ours, some patients were identified, who presented with ctDNA aberrations without any sign of clinical relapse.

More recently, Cohen et al. [24] presented a large, well-conducted study, in which a tTDS approach using a nonspecific cancer panel was adopted together with the search for plasma proteins for the early diagnosis of cancer. The authors reported poor sensitivity with such workflow in BC and focused their attention to neoplasms that are currently lacking effecting screening strategies, such as pancreatic and ovarian cancer. It has to be noted that the use of a nonbreast tTDS panel may have brought to unsatisfactory results for BC and that the purpose of that work was early noninvasive diagnosis rather than noninvasive monitoring of response and relapse and, as such, it did not have multiple time points available for assessment.

Our study presents several points of weakness: several cases were either lost to follow-up or did not present with all the time points we aimed for. Moreover, we tried to use ddPCR to detect only one potentially trackable mutation, whereas the use of multiple probes may have led to better results in terms of personalized assessment of MRD. The use of a commercial, small tTDS panel not covering key genes frequently mutated in BC such as CDH1 or the whole TP53 exonic region constitutes a strong limitation in assessing the true value of such method in the tested context and serves only as a proof-of-principle analysis of the potential of tTDS in the setting of MRD monitoring. Finally, we do not know whether finding ctDNA mutations in plasma by tTDS, not observed in biopsies of the primary tumor or identified in biopsies but not in plasma, is due to cancer subclonal emergence or to artefacts, and at present, it is difficult to prove or disprove either theory in the absence of a transversal validation method.

Nonetheless, there are several key points in our work that may be of interest for the scientific community, especially for what concerns the pitfalls and caveats of the methods we tested in a clinical practice-like setting. First, the quantity of ccfDNA in nonmetastatic patients was consistently very limited. Retrieving the expected quantity of DNA for both our assays (10 ng is recommended by Bio-Rad for ddPCR and 20 ng by Thermo Fisher for tTDS) was not feasible from a single 7.5 EDTA peripheral blood test tube in most cases. With the recent marketing of larger, ctDNA-optimized tubes, a higher yield is now realistic. Care must still be taken because there are physical limitations—no matter the ctDNA detection method used—to how many mutated molecules we may expect to find in plasma in early BC. Given the weight of a human genome (about 3.6 pg), with a method that may theoretically detect one mutated molecule in 1,000—i.e., a LOD of 0.1%—we will still need at least triple the amount of the DNA from 1,000 nuclei to stand a good statistical chance to observe one mutated DNA fragment in a thousand. We are still not aware of how many molecules may circulate in BC MRD cases, and the fact that, at best, we identified mutations in our patients' ctDNA using either ddPCR or tTDS, with a lead time of six months over clinical relapse suggests that a metastasis just below the detectable size of CT scans—around 0.5 cm—may be the smallest ctDNA-releasing lesion we could identify.

Our study has the strength of comparing head-to-head, albeit in a small case set, the two most promising methods, which are currently available for ctDNA detection. In our experience, MPS followed by the design of ddPCR-personalized assays proved to be more cumbersome than tTDS and did not lead us to more satisfying results than the latter method. Indeed, it is hardly realistic at present to imagine a clinical practice workflow for early BC patients in which MPS is followed by ddPCR probe design, validation, and use over short periods of time such as monitoring the response to NACT.

This pipeline may be used for monitoring of MRD, but is limited by the identification of one (or more) good candidate target mutation and the successful design of at least one ddPCR probe.

On the other hand, tTDS is characterized by its capability to interrogate hundreds of hotspots simultaneously without the need for optimization in each case. Provided the right conditions are met, especially the presence of a sufficient DNA yield from plasma, tTDS seems of more immediate transferability to clinical practice. With the recent possibility of designing custom tTDS panels, which include the most frequently mutated genes in BC, such as TP53, CDH1, GATA3, and PIK3CA hotspots, the use of this method may lead to an effective way to monitor the presence of MRD in a significant proportion of early BC patients.

In conclusion, our work showed that, in principle, tTDS is a promising technique for the detection of MRD in BC. Further studies should assess its use after target design optimization and by increasing the quantity of plasma to be used for ctDNA detection. Ultimately, the goal of applying tTDS in early BC is, however, to demonstrate not only its clinical validity, but rather its medical utility. This latter task may lead to effective strategies aimed at altering the course of relapsed disease when detected earlier than clinical progression, and studies directed to this purpose are strongly needed.

## Figures and Tables

**Table 1:** Demographics and pathological data of the studied cohort.

UPN	Age at diagnosis (years)	Baseline	ER	HER2	Ki-67 (%)	Pre surg stg	Surg stg	pCR
1	37	cT2(40)N1	+	−	40	cT2(35)N0	pT3N2a	No
2	63	cT2(40)N0	+	+	10	cT1(1)cN0	ympT1cN1a	No
3	39	cT2(24)N0	+	−	70	cT0N0	ypTisN0	Yes
4	73	cT2(30)N1	+	−	16	cmT1(8)bN0	ypT1bN1a	No
5	44	cmT2(24)N0	+	+	60	cT0N0	ypCR	Yes
6	55	cT2(43)N1	+	−	10	cT1(12)cN0	ypT1aN0	No
7	61	cT2(22)N0	+	−	90	cT0N0	ypCR	Yes
8	54	cT3(60)N0	+	−	10	cT0N0	ypT1cN0	No
9	44	cT3(72)N1	+	−	45	cT1(10)N0	ypT1cN1a	No
10	32	cT3(90)N1	+	−	70	cT1(12)cN0	ypT3N2a	No

UPN = unique patient number. Baseline = clinical stage at diagnosis, before NACT. Pre surg stg = clinical staging before surgery and after NACT. Surg stg = pathological staging at surgery. pCR = pathological complete response.

**Table 2:** Selected mutations at diagnosis by Comprehensive Cancer Panel™ on FFPE biopsies.

UPN	Genotype	Frequency (%)	Gene	Coding	AA change
1	CAAAT/CAAAC	18.1	TP53	c.614A>G	Tyr205Cys
2	A/G	8.82	PIK3CA	c.1625A>G	Glu542Gly
3			None detected		
4	C/T	21.12	TP53	c.158G>A	Trp53Thr
5			None detected		
6	A/G	29.7	PIK3CA	c.3140A>G	His1047Arg
7			None detected		
8	G/A	6.4	PIK3CA	c.1624G>A	Glu542Lys
9			None detected		
10	C/CA	27.53	GATA3	c.1223_1224insA	Pro409fs

Mutation profile by high-depth targeted MPS in FFPE biopsy of 10 patients: Comprehensive Cancer Panel™ identified somatic genetic alterations in three different genes TP53, PIK3CA, and GATA3 in six patients. According to the stringent selection criteria we established for personalized mutational marker selection, no somatic alterations were found in four patients.

**Table 3:** Mutational analysis by ddPCR on FFPE biopsy and on ctDNA.

Relapsed Patients (UPN)	Target mutation selected by MPS			ddPCR ctDNA sampling			
		Pretreatment	(T0)	3 months postsurgery	(T3)	Prerelapse	Relapse
1	TP 53 p.Tyr205Cys	Pos		Neg		Pos	Pos
6	PIK3CA p.His1047Arg	Pos		Neg		Neg	Neg
10	GATA3 p.Pro409fs	Pos		Neg		Neg	Neg

Nonrelapsed patients (UPN)	Target mutation selected by MPS	Pretreatment	(T0)	3 months postsurgery	(T3)	24 months postsurgery (T7)	

2	PIK3CA p.Glu542Gly	Pos		Neg			Pos
4	TP 53 p.Trp53Thr	Neg		Neg			Pos

**Table 4:** Mutational analysis by MPS and tTDS on FFPE biopsy, comparison between MPS and tTDS on FFPE biopsies samples at diagnosis.

		Target mutation selected by MPS (% frequency)	Target mutations selected by tTDS (% VAF)
		AmpliSeq CCP w1.1—Tumor-Normal pair	Oncomine TagSeq Breast v2 Liquid Biopsy w2.1—Single Sample
Relapsed patients	UPN 1	TP 53 p.Tyr205Cys (18)^#^	TP 53 p.R273H(0.3); ESR1 P.V392I (0.4); TP53 p.V157I
			(0.4); TP53 p.P82L(0.4)
	UPN 6	PIK3CA p.His1047Arg (29.7)	PIK3CA p.H1047R (23.2); Tp53 p. R282W (1.1)
	UPN 10	GATA3 p.Pro409fs (28.0)^#^	
		TP53c.919 + 1G>C p.?chr 17:7577018(43.0)	TP53c.919 + 1G>C p.?chr 17:7577018 (50.0)
	UPN 8	PIK3CA p.E542K (5.9)	PIK3CA p.E542K (1.5)

Nonrelapsed patients	UPN 2	PIK3CA p.Glu542Gly (9.0)	PIK3CA p.Glu542Gly (8.4)
	UPN 4	TP 53 p.Trp53Thr (21.0)^#^	ERBB3 p. V104M(0.5); TP53 R248W (0.3)
	UPN 5	Nondetected SNV	Nondetected SNV/ERBB2 gain
	UPN 7	TP53c.994 − 1G>C; chr17:7574034 p? (48.2)	TP53 chr17:7574034 p? (48.6); TP53 p. R273H (0.3)
			CCND1 gain
	UPN 9	Nondetected SNV	FBXW7 p.S582L (0.6)

^#^Mutation not present in Oncomine Breast cfDNA panel.

**Table 5:** Mutational analysis by tTDS on ctDNA.

Oncomine Breast liquid Biopsy v2.0 Analysis Ion Reporter: Workflow Oncomine TagSeq Breast v2 Liquid Biopsy w2.1—Single Sample				
*A*		*FFPE biopsy (% MAF)*	*Prerelapse (% MAF)*	*Relapse (% MAF)*
Relapsed patients	UPN1	TP 53 p.R273H(0,3); ESR1 P.V392I (0,4); TP53 p.V157I (0,3); TP53 p.P82L(0,4)	T4_nondetected SNV	T5_nondetected SNV
	UPN6	PIK3CA p.H1047R (23,2); Tp53 p. R282W (1,1)	T5_ PIK3CA H1047R (0,1); PIK3CA E726K (0,2); Tp53 p. R282W (0,1)	T6_PIK3CA H1047R (0,2)
	UPN 10	TP53 c.919 + 1G>C p.? chr17:7577018 (50)N,P	T5_ CCND1 loss; T7_TP53 c.919 + 1G>C p.? chr17:7577018 (0,2)N,P	T8_not available
	UPN 8	PIK3CA p.E542K (1,5)	T4_KRAS G12D (0,1)	T5_G12V(0,4); T6 _G12V (0,2)

*B*		*FFPE biopsy (% MAF)*	*Follow-up 24 months (T7) (% MAF)*	

Nonrelapsed patients	UPN2	PIK3CA p.Glu542Gly (8,4)	Nondetected SNV	
	UPN 4	ERBB3 p. V104M(0,5); TP53 R248W (0,3)	TP53 p.R248W (3,9); PIK3CA H1047R (0,6)	
	UPN 3	Nondetected SNV	Nondetected SNV	
	UPN 7	TP53 chr17:7574034 p? (48,6)N,P; TP53 p.R273H(0,3)_CCND1 gain	TP53 p.R273H (1,1); PIK3CA H1047R (0,3); KRAS p.G12A (0,4)	
	UPN 5	Nondetected SNV_ERBB2 gain	Nondetected SNV	
	UPN9	FBXW7 p.S582L(0,6)	Nondetected SNV	

Mutational analysis on ctDNA and FFPE biopsy by tTDS. A: relapsed patients and B: nonrelapsed patients.

## Data Availability

The data used to support the findings of this study are available from the corresponding author upon request.

## Abbreviations

BC: Breast cancer
MRD: Minimal residual disease
ctDNA: Circulating tumor DNA
ccfDNA: Circulating cell-free DNA
MPS: Massive parallel sequencing
NACT: Neoadjuvant chemotherapy
tTDS: Tagged targeted deep sequencing
ddPCR: Digital droplet polymerase chain reaction.
